# Estimating myocardial perfusion from dynamic contrast-enhanced CMR with a model-independent deconvolution method

**DOI:** 10.1186/1532-429X-10-52

**Published:** 2008-11-12

**Authors:** Nathan A Pack, Edward VR DiBella, Thomas C Rust, Dan J Kadrmas, Christopher J McGann, Regan Butterfield, Paul E Christian, John M Hoffman

**Affiliations:** 1 Department of Bioengineering, University of Utah, Salt Lake City, Salt Lake County, Utah, USA; 2 Utah Center for Advanced Imaging Research, Department of Radiology, University of Utah, Salt Lake City, Salt Lake County, Utah, USA; 3 Huntsman Cancer Institute, University of Utah, Salt Lake City, Salt Lake County, Utah, USA

## Abstract

**Background:**

Model-independent analysis with B-spline regularization has been used to quantify myocardial blood flow (perfusion) in dynamic contrast-enhanced cardiovascular magnetic resonance (CMR) studies. However, the model-independent approach has not been extensively evaluated to determine how the contrast-to-noise ratio between blood and tissue enhancement affects estimates of myocardial perfusion and the degree to which the regularization is dependent on the noise in the measured enhancement data. We investigated these questions with a model-independent analysis method that uses iterative minimization and a temporal smoothness regularizer. Perfusion estimates using this method were compared to results from dynamic ^13^N-ammonia PET.

**Results:**

An iterative model-independent analysis method was developed and tested to estimate regional and pixelwise myocardial perfusion in five normal subjects imaged with a saturation recovery turboFLASH sequence at 3 T CMR. Estimates of myocardial perfusion using model-independent analysis are dependent on the choice of the regularization weight parameter, which increases nonlinearly to handle large decreases in the contrast-to-noise ratio of the measured tissue enhancement data. Quantitative perfusion estimates in five subjects imaged with 3 T CMR were 1.1 ± 0.8 ml/min/g at rest and 3.1 ± 1.7 ml/min/g at adenosine stress. The perfusion estimates correlated with dynamic ^13^N-ammonia PET (y = 0.90x + 0.24, r = 0.85) and were similar to results from other validated CMR studies.

**Conclusion:**

This work shows that a model-independent analysis method that uses iterative minimization and temporal regularization can be used to quantify myocardial perfusion with dynamic contrast-enhanced perfusion CMR. Results from this method are robust to choices in the regularization weight parameter over relatively large ranges in the contrast-to-noise ratio of the tissue enhancement data.

## Background

Dynamic contrast-enhanced cardiovascular magnetic resonance (DCE-CMR) is a commonly used tool for detecting and quantifying reductions in myocardial blood flow (perfusion) in patients with coronary artery disease (CAD). The early diagnosis of CAD can provide valuable information that may affect interventional strategies in patients with ischemia [1]. In DCE-CMR perfusion studies, a paramagnetic gadolinium (Gd) complex is injected into the patient while at rest and at stress–during which a pharmacological vasodilator (adenosine) is simultaneously administered to the patient. Once the Gd is injected, it flows through the heart and temporarily distributes in the myocardium before being 'washed out' of the body. The spatiotemporal distribution of Gd within the heart can be measured dynamically and the resultant blood and tissue enhancement data can be analyzed to estimate the rate of perfusion to each region of the myocardium. A quantitative estimate of regional myocardial perfusion can provide an objective measure of the severity of myocardial injury and may help clinicians to discriminate regions of the heart that are at increased risk for myocardial infarction.

Estimates of myocardial perfusion from DCE-CMR studies have been reported using a number of different analysis methods [2-6]. Most quantitative analysis methods require that the measured blood and tissue enhancement data are mathematically deconvolved in order to estimate the system impulse response function, *h(t)*, from which myocardial perfusion can be computed. Physiologically-derived tracer kinetic models are often used to parameterize *h(t) *in the deconvolution process to ensure that the estimate of *h(t) *has a sensible physiologic interpretation. Alternatively, model-independent deconvolution, based on the central volume principle [7], can be used to estimate *h(t) *and perfusion. Model-independent analysis is relatively widely used with intravascular contrast agents and has been applied with extracellular contrast agents to quantify renal and tumor perfusion [8,9]. Also, a model-independent approach that uses B-splines and temporal regularization to parameterize *h(t) *has been developed to estimate myocardial perfusion [4,10-12].

The objective of this work is to characterize a model-independent deconvolution method in an inverse problem framework that uses iterative minimization with temporal regularization to estimate myocardial perfusion. The proposed method is general, as it does not use specific models to quantify perfusion and it does not use B-splines or other polynomials to parameterize *h(t)*. Using simulations and dynamic CMR perfusion data, we evaluate how the choice of regularization parameter, the type of regularization, and the number of iterations used in the algorithm affect estimates of myocardial perfusion. As part of this analysis, we demonstrate that the optimal regularization weight parameter in model-independent analysis is dependent on the contrast-to-noise ratio (CNR) of the measured blood and tissue enhancement data. Myocardial perfusion estimated with this method in five subjects imaged with 3 T CMR was compared with a current standard for quantitative myocardial perfusion, dynamic ^13^N-ammonia positron emission tomography (PET).

## Methods

### Model-Independent deconvolution using iterative minimization and temporal regularization

#### The *central volume principle *and model-independent analysis

Zierler et al. developed a theoretical framework using the central volume principle for measuring fluid flow into regions of tissue [7]. For biological systems that involve fluid flow into a finite volume of tissue, the impulse response function of the system, *h(t)*, provides a quantitative measure of the rate of flow to the tissue. According to the central volume principle, the maximum amplitude of *h(t) *is directly related to the rate of perfusion to the region of interest, independent of physiologic parameters such as the distribution and permeability of the vasculature within the tissue. The primary assumption of the central volume principle is that the impulse response function can be described by a stationary, linear system with a single input and a single output. In DCE-CMR perfusion studies, the measured LV blood enhancement signal, C_bld_(t), represents the system input and the measured myocardial tissue enhancement signal, C_tis_(t), represents the system residue. Thus, *h(t) *and regional myocardial perfusion can be estimated by directly deconvolving C_bld_(t) and C_tis_(t).

Jerosch-Herold pioneered the use of model-independent deconvolution for estimating myocardial perfusion and has used this approach in several recent studies [4,10-12]. In each of those studies, *h(t) *was parameterized as a sum of weighted B-spline functionals and temporal regularization was used to constrain the estimate of *h(t)*. Here we present an alternative approach for quantifying myocardial perfusion with model-independent analysis, by formulating a cost function in which *h(t) *is estimated directly with temporal regularization [13]. Eq (1) shows the cost function that is minimized in this study.

(1) ||*C*_*bld*_(*t*) ⊗ *h*(*t*) - *C*_*tis*_(*t*)||^2 ^+ *λ*^2 ^||∇ *h*(*t*)||^2^

Here C_bld_(t) and C_tis_(t) represent the measured blood and regional tissue enhancement curves of the LV, respectively; *h(t) *is the system impulse response function; λ is the regularization weight parameter; ∇ is the temporal gradient operator; and ||*|| denotes the Euclidean norm (L_2_-norm) operator. The temporal regularizing constraint included in the cost function balances the accuracy of the estimates of C_tis_(t) and the temporal smoothness of *h(t)*. Myocardial perfusion estimates are calculated from the maximum amplitude of *h(t)*, scaled by the inverse of the sampling rate of the perfusion images and the specific gravity of the myocardial tissue (SG_myo_): Flow = max [h(t)]/(dt*SG_*myo*_*) *[7].

#### Determination of the regularization type and parameter

Without the inclusion of regularization, the cost function minimization problem shown in Eq (1) may not be unique. Noise and other inaccuracies in the measured CMR perfusion data can exacerbate this problem. Regularization techniques have been developed to condition ill-posed and non-unique problems such as these by imposing a penalty on the solution when specified conditions are not satisfied [14,15]. The type of regularization used can be chosen based on the class of problem being solved or on *a priori *assumptions about the expected solution to the problem. Our proposed implementation readily accommodates multiple constraints that can help regularize the solution of *h(t) *in the minimization process. In this work we have chosen to include a first-order temporal gradient constraint with a Euclidean norm (L_2_-norm) operator to ensure smoothness in the estimate of *h(t)*. Spatial gradients could also be incorporated, and have been explored recently in the context of DCE tumor studies using compartment modeling [16].

To ensure that there is a good trade-off between the goodness-of-fit of the estimated C_tis_(t) curves and the overall smoothness of *h(t)*, L-curve analysis [17] was used to determine the regularization weight parameter, λ, in all five subjects. With L-curve analysis, the log of the regularization norm, ||∇ h(t)||, is plotted versus the log of the residual norm, ||C_bld_(t)⊗ h(t) - C_tis_(t)||, for different values of λ. The region of maximum curvature on the L-curve represents the value of λ for which there is an optimal trade-off between the goodness-of-fit of the estimated C_tis_(t) curve and the temporal smoothness of *h(t)*.

Because the value of the regularization weight parameter is dependent on the CNR of the measured blood and tissue enhancement data, a different λ value was calculated for each subject in this study. L-curve analysis was used to determine the optimal λ values for 15 representative regions of tissue enhancement from the 6-region data in all five subjects at rest (three regions in five LV slices; one slice per subject). From these λ values, an average λ value was also calculated. Perfusion estimates were then computed for the 6-region data using the optimal λ values for each region and using the average λ value for all the regions, to determine whether there was a significant difference between using an optimal versus a near optimal λ value in model-independent analysis. Because the CNR of the data measured in all five subjects in this study was similar, an average λ value was used to estimate myocardial perfusion in all five subjects from the 6-region data (at rest and stress). Perfusion estimates computed using the average λ value varied by less than 1% from perfusion estimates computed using the optimal λ for each subject.

Gradient descent minimization [18] was used in the iterative minimization process to compute *h(t)*, with an initial estimate of constant *h(t)*, *h(t) *= 0.001. The iterative minimization process was repeated until the L_2_-norm curve-fit error–the difference between the measured C_tis_(t) data and the estimated C_tis_(t) data, computed by convolving the measured C_bld_(t) with the estimate of *h(t)*–was less than 5% or until n = 600 iterations were completed. A fixed step size of γ = 1 × 10^-9 ^was used in the minimization process for all five subjects in the study. In most cases, the algorithm rapidly converged on an estimate of *h(t) *that was within 5% of a steady-state solution, while avoiding oscillations and non-convergent estimates of *h(t)*.

### Simulations and CMR patient studies

#### Acquisition of magnetic resonance image data

Five male volunteers (age: 49 ± 17 yrs) were imaged with 3 T CMR (and dynamic ^13^N-ammonia PET) in this study. The CMR and PET imaging protocols were approved by the University of Utah Institutional Review Board and informed consent was obtained from each subject prior to imaging. One of the subjects had a heart transplant 15 months prior to this study; the other four subjects were healthy with no cardiovascular risk factors. Four of the subjects were imaged first with CMR and then with PET approximately one month later. The fifth subject was imaged with PET first and then with CMR six months later. All the subjects were asked to abstain from caffeine for at least 12 hours prior to the imaging studies and imaging was always performed in the morning.

The dynamic CMR perfusion data was acquired with a Siemens Trio 3 T CMR scanner (Siemens Medical Systems, Erlangen, Germany) using a gradient echo TurboFLASH pulse sequence with saturation recovery magnetization preparation and a linear order phase encoding scheme. While at rest, two subjects were imaged after being given a 0.025 mmol/kg dose of Gd-DTPA (Omniscan; Amersham Health Inc., Princeton, NJ). Three other subjects were given a low-dose (0.017 ± 0.005 mmol/kg) bolus injection of Gd-BOPTA (Multihance; Bracco Diagnostics Inc., Princeton, NJ). The change in contrast agent was due to a change in internal policy [19,20]. In each patient study, the contrast agent bolus was followed by a saline flush of 15 ml using a Medrad Spectris Solaris MR power injector (Medrad, Inc., Indianola, PA) via an antecubital vein. Also, for each subject imaged, imaging parameters were selected in order to acquire at least three short axis (SA) slices of the left ventricle (LV) during every heart beat. Typical imaging parameters were: Saturation recovery time~100 ms, TR~2 ms, TE~1 ms, flip angle~12°, slice~8 mm, FOV~360 × 270, resolution~1.9 mm × 2.4 mm, with GRAPPA (R = 1.7).

Dynamic stress images were acquired approximately 10 minutes after the dynamic rest scans with the same sequence and parameters described above. During imaging, a continuous infusion of adenosine (140 μg/kg/min) (Adenoscan; Astellas Pharma US, Inc., Deerfield, IL) was administered to each subject via an antecubital vein to induce vasodilation. Approximately three minutes after the start of the adenosine infusion, a 0.025 mmol/kg dose of Gd-DTPA followed by a saline flush of 15 ml was given to two of the subjects, while images were acquired. The remaining three subjects were given a low-dose (0.023 ± 0.002 mmol/kg) bolus injection of Gd-BOPTA followed by a 15 ml saline flush. While all the rest and stress injections were assumed to be in the range of linear signal enhancement, a reduced dose was given at rest to minimize the potential effects of saturation in the later injection of Gd given at stress. All injections of Gd and saline were administered at 5 cc/s. During the dynamic rest and stress imaging, most patients held their breath for approximately 10–20 seconds during the first-pass of contrast agent through the LV and then breathed shallowly for the remainder of the scan. Approximately one minute of image data was obtained in all the subjects. Table 1 shows the average heart rate and blood pressure measurements taken at rest and stress for the five subjects in the study.

**Table 1:** Average rest and stress heart rate (HR) and blood pressure (BP) measurements recorded from each subject during the CMR and PET imaging studies.

	**Subject 1**	**Subject 2**	**Subject 3**	**Subject 4**	**Subject 5**
**CMR**					

Baseline HR (bpm)	81	56	64	43	62
Baseline BP (mmHg)	168/100	112/76	143/73	153/75	98/50
Max. Stress HR (bpm)	88	90	90	68	75
Max. Stress BP (mmHg)	182/93	-	158/71	206/85	128/80

**PET**					

Baseline HR (bpm)	80	56	56	43	56
Baseline BP (mmHg)	110/70	141/82	129/78	148/64	98/50
Max. Stress HR (bpm)	91	99	80	60	64
Max. Stress BP (mmHg)	120/72	159/92	122/71	164/85	112/50

Blood pressure measurements from the CMR studies were taken using a leg cuff. Blood pressure measurements from the PET studies were taken using an arm cuff.

#### Magnetic resonance image processing

All CMR post-processing was performed using Matlab Version 7.2 (The MathWorks Inc. Natick, MA). The image frames for each slice of the LV were manually registered for in-plane rigid body motion and endocardial and epicardial contours were manually drawn to segment the LV myocardium for perfusion analysis. Each myocardial slice was divided into six equiangular sections and each section was normalized to the mean pre-contrast signal of the entire LV–which was assumed to have a uniform signal intensity–to correct for regional CMR coil sensitivity [21,22]. Dynamic tissue enhancement curves, C_tis_(t), were then obtained from each LV region by subtracting off the mean value of the pre-contrast pixels in that region. A single dynamic blood enhancement curve, C_bld_(t), was obtained from the mean change in signal intensity of a manually selected region of the LV blood pool in a mid-basal SA slice. Because the C_bld_(t) curve used in the deconvolution process was obtained from one LV slice, the C_tis_(t) curves from adjacent LV slices were scaled to correct for variations in the pre-contrast signal in the myocardium that may have resulted from different coil sensitivity profiles in the adjacent slices. To reduce overestimation effects caused by noise in the pre-contrast frames of C_tis_(t) and C_bld_(t), the pre-contrast data prior to the onset of blood and tissue enhancement was not used in the curve-fitting process. These same steps were repeated for the pixelwise C_tis_(t) data analysis.

For the image post-processing analysis it was assumed that for the relatively low doses of contrast agent used, the change in signal intensity of C_bld_(t) and C_tis_(t) from the CMR perfusion images was linearly proportional to the change in local contrast agent concentration within the LV blood pool and the myocardial tissue [23,24]. Image acquisition times for each frame were obtained from the scanner in order to account for each patient's variable heart rate or missed beats during the scan. This time-stamp correction process has been shown to ensure more accurate estimates of perfusion in dynamic contrast-enhanced CMR studies [25]. Also, prior to deconvolution of the C_bld_(t) and C_tis_(t) curves in each region of the myocardium, a global time delay was estimated for each LV slice. The global time delay was the mean delay time between LV blood pool enhancement and local tissue enhancement in the myocardium and was used as a fixed parameter when estimating regional and pixelwise perfusion in the LV myocardium [26]. Finally, estimates of myocardial perfusion were computed assuming the LV myocardium has a specific gravity of 1.06 g/ml.

#### The effects of noise on estimates of myocardial perfusion

Because DCE-CMR perfusion images can have a higher spatial and temporal resolution than other imaging modalities, it may be better able to discriminate small regions of reduced perfusion in the myocardium. However, it is unknown how well the proposed model-independent analysis method estimates myocardial perfusion when a non-optimal regularization parameter is used in the analysis or when very noisy curves such as those from single pixels in low-dose studies are evaluated.

For this analysis, several different sized regions of tissue enhancement in each subject were selected and an optimal λ value was determined for each region using L-curve analysis. This evaluation is important because the choice of λ using L-curve analysis is dependent on the CNR of the blood and tissue enhancement data. For each subject, tissue enhancement regions comprised of 2000, 1000, 500, 100, 50, 20, 5, and 1 pixel–each with a different CNR–were used. The optimal λ values for each of the regions were plotted versus the corresponding CNR of the enhancement data to determine whether a nonlinear relationship exists between the regularization weight parameter and the CNR of the data. CNR was computed as the peak signal enhancement in each tissue region, divided by the standard deviation of the pre-contrast signal intensity in that same region.

#### The effect of λ on 6-region perfusion estimates

To evaluate how large deviations above and below the optimal λ value affected the aggregate perfusion estimates, several λ values, above and below the previously determined average λ value, were used to over-regularize and under-regularize the tissue enhancement data when model-independent deconvolution was performed. The mean perfusion estimates and the coefficients of variation (CV) of the perfusion estimates from the 6-region data in all five subjects in the study (at rest) were plotted versus the changes in λ.

#### The effect of λ on pixelwise perfusion estimates

Because the CNR of pixelwise enhancement data may vary widely within a single LV slice of the myocardium, the optimal λ values from separate pixel enhancement curves may also be significantly different. As mentioned above, perfusion may be overestimated if the noisy pixelwise data is under-regularized with an inappropriate average λ value. To evaluate the use of an average λ value for pixelwise enhancement data, perfusion estimates were computed in each of the 40 single pixel regions described above using the optimal λ value for each curve, and using different average λ values for each subject, and using a single average λ value for all five of the subjects combined. The perfusion estimates computed using optimal λ values for each of the 40 pixel regions were plotted versus the perfusion estimates computed using one average λ value for each subject or when using a single global pixelwise λ value for all of the subjects.

Pixelwise perfusion estimates (mean ± standard deviation) were compared to 6-region perfusion in each LV slice in all five subjects. Pixelwise perfusion estimates were computed by deconvolving the tissue enhancement data, C_tis_(t), from each image pixel in each LV slice from the measured C_bld_(t) data. In a similar manner as the 6-region analysis, optimal λ values were determined using L-curve analysis for eight representative pixelwise enhancement curves in each of the five subjects at rest (eight pixels in five LV slices; one slice per subject). An average pixelwise λ value was also calculated. Aggregate pixelwise perfusion estimates were computed using the average pixelwise λ value. Pixelwise perfusion estimates were also computed using the average 6-region λ value to determine the effects of using under-regularizing λ value.

### Comparison with dynamic ^13^N-ammonia PET imaging

#### Acquisition of PET data

On a separate day from the CMR scans, dynamic rest and stress PET images were acquired in the same five subjects with a GE Advance PET scanner (GE Medical Systems, Waukesha, WI) operated in 2D mode. A 10 minute transmission scan was performed using a ^68^Ge rod source to measure photon attenuation and to ensure that the heart was in the field of view. Each subject was then given a ~20 mCi injection of ^13^N-ammonia over 30 seconds followed by a saline flush of 15 ml at 5 cc/s. Dynamic rest images were acquired for 20 minutes. The temporal sampling protocol was: 12 × 5 s, 6 × 10 s, 6 × 30 s, 5 × 1 min, 5 × 2 min. Approximately one hour after the start of the rest scans (the half-life of ^13^N-ammonia is 9.97 minutes), dynamic stress imaging was started using the same time sampling protocol as at rest with another ~20 mCi injection of ^13^N-ammonia, while a continuous infusion of adenosine (140 μg/kg/min) was administered for six minutes [27].

#### PET image processing

Prior to reconstruction, the PET images were corrected for photon attenuation and scatter. The images were reconstructed to a voxel size of 4.3 mm × 4.3 mm × 4.3 mm using a filtered back-projection algorithm with a Hanning window cutoff of 1.6 cycles/cm. The image frames from the rest and adenosine stress studies were manually registered and three SA slices of the LV myocardium were selected to match those from the CMR studies for perfusion analysis. Within each SA slice, a blood pool region of the LV was manually selected and the myocardium was divided into six equiangular regions, similar to those used in the CMR studies. The resulting ^13^N-ammonia time-activity curves from the blood and tissue regions were fit to a 3-compartment model, which included the effects of radioactive decay [28,29]. The expression for the ^13^N activity concentration, C(t), in each region of myocardium is shown in Eq (2).

(2) *C*(*t*) = *V*_*b*_*C*_*b*_(*t*) + (1 + *V*_*b*_)*C*_*t*_(*t*)

In Eq (2), Vb is the total fraction of the blood volume in each region of tissue, C_b_(t) is the ^13^N activity concentration in the blood, and C_t_(t) is the ^13^N activity concentration in each myocardial tissue. Eq(3) shows the expansion of C_t_(t) in terms of the kinetic rate constants (k_1_, k_2_, and k_3_), a radioactive decay constant, λ, and a metabolite-corrected arterial input function, b(t), where ⊗ is the convolution operator [30,31].

(3) $$C_{t}(t)=\left[\frac{k_{1}k_{3}}{k_{2}+k_{3}}e^{-\lambda t}+\frac{k_{1}k_{2}}{k_{2}+k_{3}}e^{-(k_{2}+k_{3}+\lambda )t}\right]⊗b(t)$$

In Eq (3), b(t) is the arterial input function which represents the fraction of unmetabolized and freely exchangeable ^13^N-ammonia available in the blood, C_b_(t), at time *t*. For this study, metabolite correction was performed based on average values reported in the literature [30,31].

#### Statistical comparison of myocardial perfusion using CMR and PET

Aggregate rest and stress myocardial perfusion estimates (mean ± standard deviation) and the myocardial perfusion reserve (MPR)–the ratio of hyperemic to resting perfusion–are reported for the five subjects imaged in the CMR and PET studies. For each subject, perfusion estimates were computed for three coronary artery territory regions in three LV slices, based on the American Heart Association (AHA) 16-segment model [32]. CMR perfusion estimates were computed using the proposed model-independent analysis method and PET perfusion estimates were computed using dynamic ^13^N-ammonia PET with 3-compartment modeling [28,29]. Linear regression was performed and a paired Student's t-test was used to evaluate the significance of the correlation between the perfusion estimates using CMR and PET from all the regions in the 16-segment model and in the territorial AHA regions. Bland-Altman analysis was used to show whether there was bias in the CMR and PET perfusion estimates.

## Results

**Figure 1:**
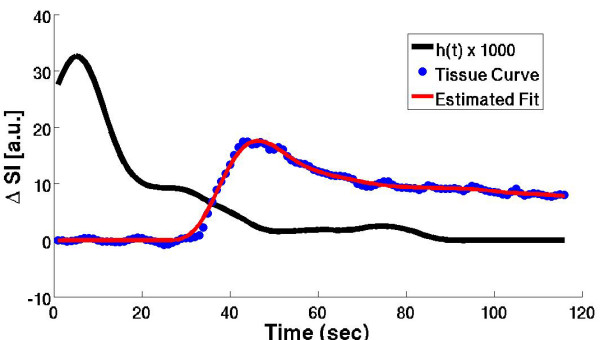
A typical *h(t) *curve computed using the proposed model-independent deconvolution method overlaid on the corresponding tissue enhancement curve, C_tis_(t), and the fitted C_tis_(t) curve computed using *h(t)*, for one subject in the study.

Figure 1 shows a typical *h(t) *curve computed using the proposed model-independent deconvolution method overlaid on the corresponding measured tissue enhancement curve, C_tis_(t), and the estimated tissue enhancement curve computed using *h(t)*, for one subject in the study. From the plot, it is noted that after a brief time delay, while the contrast agent distributes in the myocardium, *h(t) *begins with an initial maximum amplitude and then approximates a monotonically decaying function similar to estimates of *h(t) *used in other model-based deconvolution methods such as 2-compartment modeling or Fermi function modeling. From the central volume principle, the maximum amplitude of *h(t) *is related to the rate of perfusion in the tissue: *Flow *= max [h(t)]/(dt*SG_*myo*_*) *[7].

**Figure 2:**
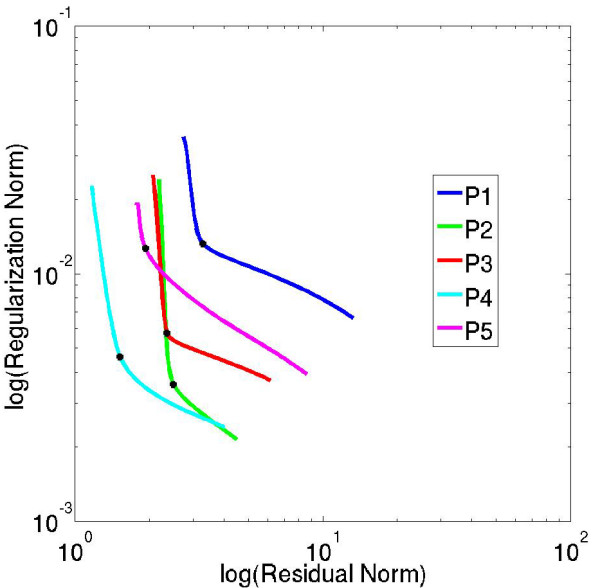
**L-curve plots for each of the five subjects in the study.** Each of the L-curves was computed for one representative region of the 6-region data in each of the five subjects at rest. These L-curves were typical of the L-curves computed from multiple regions of tissue enhancement in all five subjects. The dark marker at the region of maximum curvature on each L-curve represents the optimal value of λ that provides a near optimal balance of the goodness-of-fit of the estimated tissue enhancement curves and the temporal smoothness of *h(t)*. The optimal 6-region λ values for the five subjects were: λ = 0.032, 0.032, 0.042, 0.028, 0.02.

Figure 2 shows one L-curve plot for each of the five subjects in the study. Each of the L-curves shown in Figure 2 was computed for one representative region of the 6-region data in each of the five subjects at rest. These L-curves were typical of the L-curves computed from multiple regions of tissue enhancement in all five subjects. The dark marker at the region of maximum curvature on each L-curve represents the optimal value of λ that provides a near optimal balance of the goodness-of-fit of the estimated tissue enhancement curves and the temporal smoothness of *h(t)*, when six regions of interest per slice were used. The five optimal regional values of λ for the subjects imaged in the study ranged from 0.02 to 0.042. Subsequently, an average regularization weighting parameter of λ = 0.03 was used for the perfusion analysis of the 6-region data for all the subjects in the study.

Perfusion estimates (at rest and stress) computed using the single average λ value varied by ~1% from perfusion estimates computed using the optimal regional λ values for each subject. This small variation in perfusion estimates demonstrates the robustness of the choice of λ in the regularization process. In the iterative minimization process, fewer than 200 iterations were required for most of the CMR datasets to estimate *h(t) *with an L_2_-norm curve-fit error less than 5%. For the cases in which the L_2_-norm curve-fit error never reached a steady-state value less than 5%, a maximum of 600 iterations was used.

**Figure 3:**
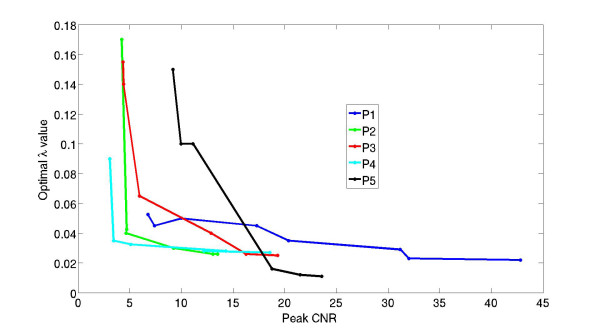
**A plot of the optimal λ values computed using L-curve analysis versus the CNR of measured tissue enhancement data in large uniform regions of enhancement down to data measured in an individual pixel in each of the five subjects.** For each subject there is a nonlinear relationship between the optimal regularization weight parameter and the CNR of the measured enhancement data. And although the correlation between these two variables is different for each subject, the figure illustrates the importance of determining an optimal λ value according to the CNR of the measured data.

**Figure 4:**
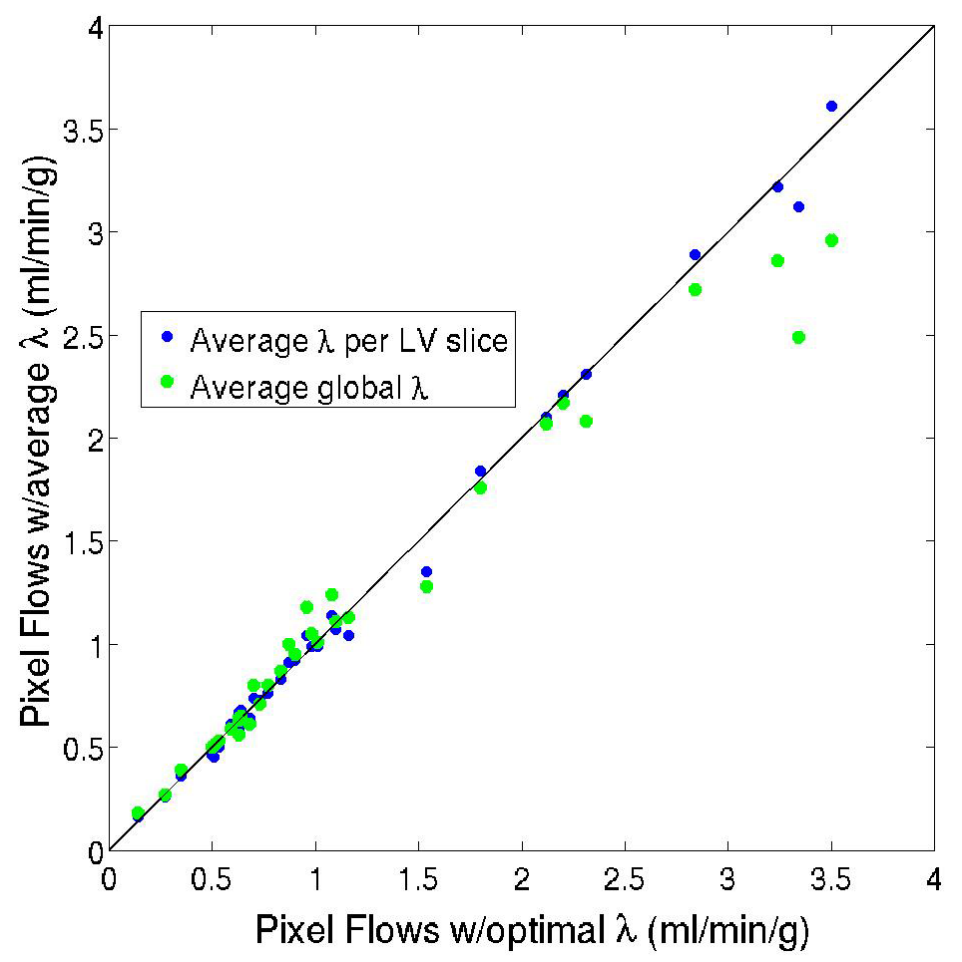
**A scatter plot of perfusion estimates in 40 single pixel regions of the myocardium (eight pixels in five LV slices; one slice per subject) when using the optimal λ value for each pixel region separately versus using one average λ value for each subject or when using a single average λ value for all 40 pixel enhancement regions.** Perfusion estimates using five average λ values or a single average λ value for all the subjects were not significantly different than the perfusion estimates computed using optimal λ values for each pixel region separately (p = 0.54 and p = 0.14, respectively).

**Figure 5:**
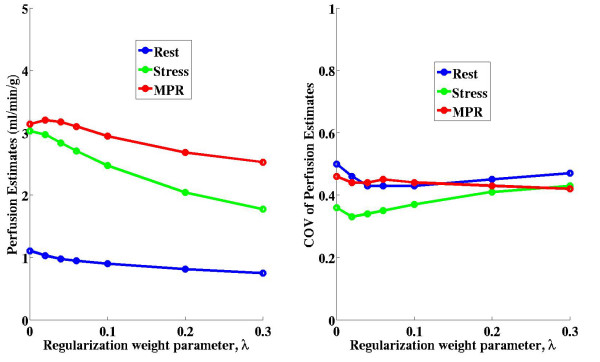
**Plots of the mean perfusion estimates (left) and the coefficients of variation (CV) (right) of perfusion estimates from all five subjects in the study versus changes in the regularization weight parameter, λ.** The average optimal λ value for these perfusion estimates was λ = 0.03. While there may be relatively large changes in perfusion estimates across a broad range of λ values, small deviations from the optimal λ value result in insignificant changes in mean perfusion estimates. This demonstrates that model-independent analysis is relatively insensitive to small deviations from the optimal regularization parameter. The coefficients of variation of the perfusion estimates remained nearly constant over a large range of λ values.

Figure 3 shows a plot of the optimal λ values computed using L-curve analysis for all five subjects in the study versus the CNR of the measured tissue enhancement data in large uniform regions of enhancement down to enhancement data measured in individual pixels. For each subject there is a nonlinear relationship between the optimal regularization weight parameter and the CNR of the measured enhancement data. And, although the correlation between these two variables is different for each subject, the figure illustrates the range of optimal λ values between subjects. The robustness of perfusion estimates to non-optimal λ values is evaluated in Figure 4 and Figure 5.

Figure 4 shows a scatter plot of perfusion estimates in 40 single pixel regions of the myocardium (eight pixels in five LV slices; one slice per subject) when using the optimal λ value for each pixel region separately versus using one average λ value for each subject or when using a single average λ value for all 40 pixel enhancement regions. Perfusion estimates using a subject specific λ value or a single average λ value for all the subjects were not significantly different than the perfusion estimates computed using optimal λ values for each pixel region separately (p = 0.54 and p = 0.14, respectively).

Figure 5 shows plots of the mean perfusion estimates (left) and the coefficient of variation (right) of perfusion estimates from all five subjects in the study versus changes in the regularization weight parameter, λ. The average optimal λ value for these perfusion estimates was λ = 0.03. While there may be relatively large changes in perfusion estimates across a broad range of λ values, small deviations from the optimal λ value resulted in insignificant changes in mean perfusion estimates. This demonstrates that model-independent analysis is relatively insensitive to deviations from the optimal regularization parameter. The coefficient of variation of the perfusion estimates remained nearly constant over a large range of λ values.

### Regional perfusion estimates

**Figure 6:**
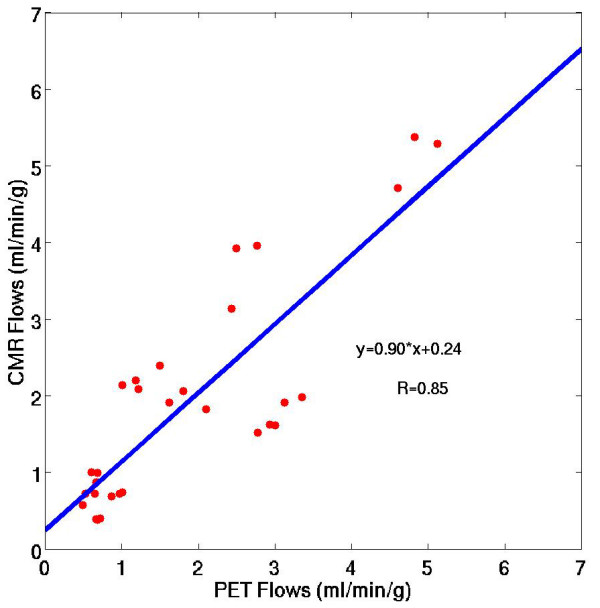
A scatter plot of the mean rest and stress myocardial perfusion estimates from 3 T CMR and dynamic ^13^N-ammonia PET for three coronary artery territories of the LV in all five subjects in the study.

**Figure 7:**
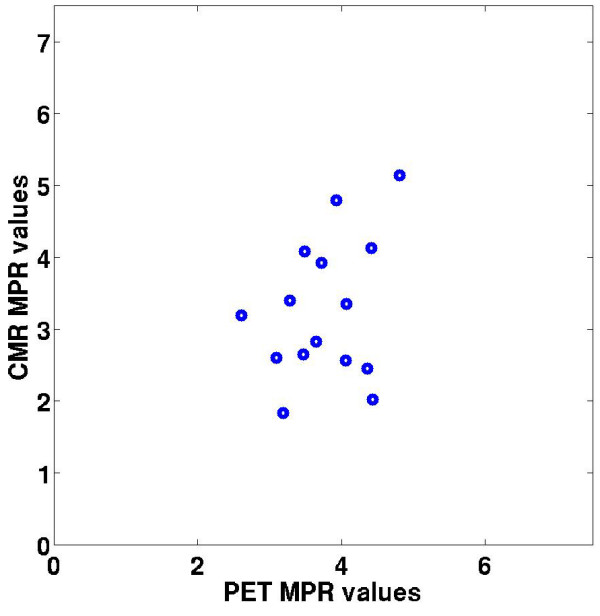
**A plot of the CMR and PET MPR values for all five subjects in the study for three coronary artery territory regions of the LV myocardium.** The mean MPR for 3 T CMR and PET were 3.2 ± 1.7 and 3.7 ± 0.7, respectively.

**Figure 8:**
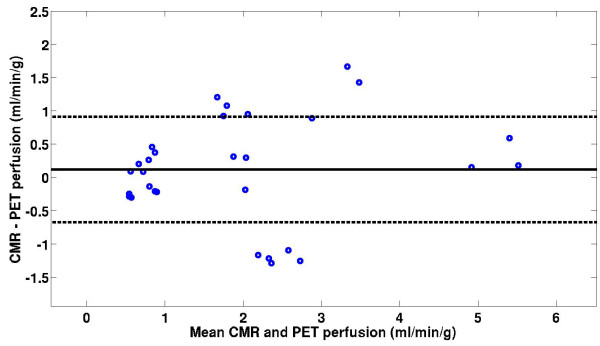
A Bland-Altman plot of regional CMR and PET perfusion estimates.

Figure 6 shows a scatter plot of the mean rest and stress myocardial perfusion estimates from 3 T CMR and dynamic ^13^N-ammonia PET for three coronary artery territories of the LV in all five subjects in the study. At rest and stress, the regional perfusion estimates using 3 T CMR were 1.03 ± 0.76 ml/g/min and 2.97 ± 1.59 ml/g/min, respectively. The corresponding perfusion estimates at rest and stress using dynamic ^13^N-ammonia PET were 0.80 ± 0.24 ml/g/min and 3.04 ± 1.14 ml/g/min. The combined rest and stress perfusion estimates were not significantly different between CMR and PET (p = 0.42) for coronary artery territorial regions of the myocardium. Similarly, the perfusion estimates were not significantly different between CMR and PET (p = 0.11) in the individual 16-segment regions of the myocardium. Figure 7 shows a plot of the MPR values for CMR and PET imaging in the same three AHA coronary artery territory regions [32]. The mean MPR for 3 T CMR and PET were 3.2 ± 1.7 and 3.7 ± 0.7, respectively. The best-fit linear regression between 3 T CMR and PET perfusion estimates was y = 0.90x + 0.24 (r = 0.85). Table 2 gives a summary of the aggregate rest and stress perfusion estimates and the MPR estimates using the model-independent analysis method with 3 T CMR and PET. Figure 8 shows a Bland-Altman plot indicating that CMR perfusion estimates had a mean overestimation of 0.12 ml/min/g versus PET perfusion estimates.

**Table 2:** Aggregate rest and stress perfusion estimates and MPR values for all five subjects imaged with 3 T CMR and dynamic ^13^N-ammonia PET.

	**Rest Perfusion (ml/min/g)**	**Stress Perfusion (ml/min/g)**	**MPR**
**CMR**	**1.03 ± 0.76**	**2.97 ± 1.59**	**3.2 ± 1.7**
**PET**	**0.80 ± 0.24**	**3.04 ± 1.14**	**3.7 ± 0.7**

These results were computed from the mean perfusion estimates in three coronary artery territory regions in three short-axis slices of the LV of each subject.

### Pixelwise perfusion estimates

**Figure 9:**
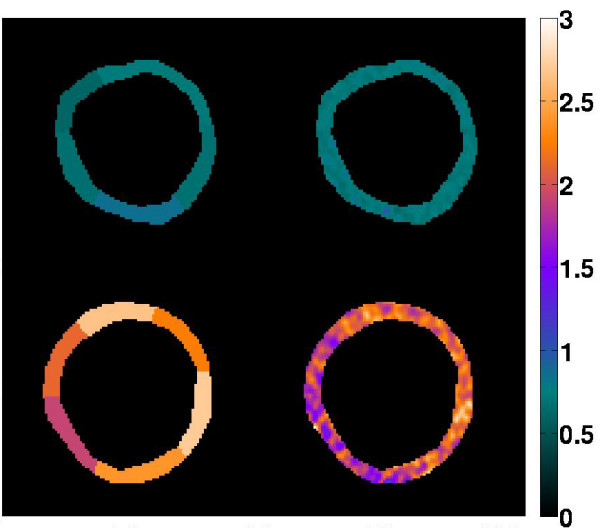
**6-region and pixelwise perfusion maps (left and right columns, respectively) at rest and stress (top and bottom rows, respectively) in one LV slice from one representative subject in the study.** The scale is in ml/min/g.

Aggregate pixelwise perfusion estimates at rest and stress were comparable to the 6-region perfusion estimates for all five subjects in the study (6-region: 1.0 ± 0.8 ml/min/g and 3.0 ± 1.6 ml/min/g versus pixelwise: 1.1 ± 0.9 ml/min/g and 2.9 ± 2.3 ml/min/g). For this comparison, different λ values were used for the 6-region data and the pixelwise data, according to the CNR of the enhancement data. The CNR of the regional enhancement data in all five subjects was 17.3 ± 8.5 and the CNR of the pixelwise enhancement data was 4.8 ± 2.0. Figure 9 shows 6-region and pixelwise perfusion maps (left and right columns, respectively) at rest and stress (top and bottom rows, respectively) in one LV slice from one representative subject in the study. When the average 6-region λ value was used for the pixelwise C_tis_(t) data, aggregate pixelwise perfusion estimates from all five subjects were 25% higher than the 6-region perfusion estimates. This overestimation was partially due to using a non-optimal λ value that under-regularized the noisy pixelwise enhancement data.

## Discussion

A fully model-independent deconvolution method that uses iterative minimization and temporal regularization has been developed and evaluated for estimating myocardial perfusion in DCE-CMR perfusion studies. Temporal regularization with a first-order gradient constraint and a Euclidean norm (L_2_-norm) operator was used to ensure temporal smoothness in the estimate of *h(t)*. The regularization overcomes the non-uniqueness of the problem solution and ensures that *h(t) *has a sensible interpretation that is not dominated by noise. This model-independent analysis method is general, as it does not rely on the assumptions of model-based analysis, which may oversimplify the kinetics of contrast agent transfer in the myocardium. And although this iterative method may not be as fast as linearized methods for estimating perfusion [4], it only requires 2–3 seconds to estimate perfusion in six regions in each LV slice (in Matlab), which is fast enough for practical use in most clinical perfusion studies.

Though a direct comparison of this proposed model-independent method and a B-spline method [4] was not the focus of this study, in our preliminary work we did compare our results with those obtained using a parameterized B-spline method. For those comparisons, the maximum amplitudes of the *h(t) *curves in 16 regions of the heart in all five subjects were not significantly different for the two methods (p = 0.16). Since our implementation of the B-spline method used truncated singular value decomposition (SVD), rather than generalized SVD as it has been published [4], it is not known how similar the results from our method and the original B-spline method are. In general, we found that the iterative method using a model with a larger number of degrees of freedom and the B-spline model, which assumes a more specific parameterization of *h(t)*, could both adequately represent the tissue enhancement data and give results similar to the widely accepted gold standard dynamic PET for non-invasively quantifying myocardial perfusion.

### Comparison of perfusion estimates from CMR and PET

Myocardial perfusion estimates using the proposed model-independent method with 3 T CMR perfusion data correlated with perfusion estimates from dynamic ^13^N-ammonia PET (y = 0.90x + .24, r = 0.85). These perfusion estimates are comparable to published results in three pigs (~1 ml/min/g and ~3.5 ml/min/g for rest and stress, respectively), in which a slightly higher dose of contrast agent (0.04 mmol/kg) and model-independent analysis with B-splines was used [4]. More recently, another group compared perfusion estimates from CMR and PET in humans using an even larger dose of contrast agent (0.08 mmol/kg) with a calibrated inversion recovery TurboFLASH pulse sequence [33]. Using this method, they found rest and stress K^trans ^estimates to be slightly lower than the perfusion estimates reported here. However, it is difficult to directly compare results from that study because of the different pulse sequence used, the larger dose of Gd, and because compartment modeling was used to estimate K^trans^, which is an index of blood flow that is often corrected by an assumed extraction fraction.

The relatively small differences in perfusion estimates between the 3 T CMR studies and the dynamic PET studies may partially be due to nonlinear variations in CMR signal enhancement versus the doses of contrast agent used in the studies. This nonlinear relationship between changes in image signal intensity and contrast agent concentration at high concentrations of Gd has been widely researched [23,24]. Some groups have concluded that the assumption of linearity in the measured C_bld_(t) data may only be valid for contrast agent doses as low as 0.005–0.01 mmol/kg [34-37]. Other groups have demonstrated that contrast agent doses as high as 0.025–0.04 mmol/kg can be used for quantifying myocardial perfusion in DCE-CMR studies [4,5,38], though this depends on the specific imaging sequence used and imaging parameters such as the saturation recovery time. Doses of ~0.017 mmol/kg for Gd-BOPTA and ~0.025 mmol/kg for Gd-DTPA were used here.

Another possibility regarding the higher values of CMR perfusion versus PET may be due to the use of 3-compartment modeling to estimate perfusion with PET. The 3-compartment model explicitly accounts for the fraction of signal enhancement in C_tis_(t) caused by the myocardial vasculature, Vb, while model-independent analysis does not. The fact that Vb is also used in 3-compartment modeling of the PET data to correct for the (large) partial volume effects of LV blood pool enhancement within the myocardium may also have effects.

The aggregate CMR MPR was 16% less than the PET MPR (3.2 ± 1.7 for CMR versus 3.7 ± 0.7 for PET), which is in somewhat better agreement than the perfusion estimates at rest. While the differences in absolute measures of rest and stress perfusion may be due to the small number of subjects imaged in the study, the similar MPR values for CMR and PET may partially be due to the cancellation of systematic effects that occur at rest and stress [39]. Furthermore, a reduced CMR MPR compared to PET has also been reported by other groups [33,40,41]. In those studies the mean CMR MPR values in healthy subjects ranged between 2.1 and 2.5, while the mean PET MPR values ranged between 2.7 and 4.3.

### Effects of the regularization weight parameter on perfusion estimates

Estimates of myocardial perfusion using model-independent analysis are dependent on the choice of the regularization weight parameter, which increases nonlinearly to handle large decreases in the CNR of the measured tissue enhancement data. This result is illustrated in Figure 3, and was further demonstrated by using the optimal λ value from the 6-region analysis to estimate perfusion in the much noisier pixelwise enhancement data. When the λ value from the 6-region analysis was used with the low CNR pixelwise data, perfusion estimates were 25% higher than the 6-region flows due to the under-regularization of the noisy pixelwise data. When an average optimal pixelwise λ value was used, the aggregate perfusion estimates were similar to those in the 6-region analysis. This finding is unique to model-independent analysis. Other model-based deconvolution methods such as compartment modeling or Fermi function analysis do not require model parameter tuning depending on the CNR of the measured enhancement data.

When the regularization weight parameter was increased slightly above or below the optimal λ value from L-curve analysis, the mean aggregate perfusion estimates only slightly decreased and increased, respectively (see Figure 5). Similarly, the coefficient of variation of the aggregate perfusion results remained nearly constant across a broad range of λ values. These findings suggest that estimates of regional perfusion are relatively insensitive to small variations (and even large variations) in the optimal λ value and that the large standard deviations in resting perfusion estimates are not likely due to over-regularization or under-regularization of the enhancement data.

For pixelwise enhancement data with a relatively wide range of CNR values, an average λ value may be suitable for estimating perfusion. As shown in Figure 4, perfusion estimates in 40 pixel enhancement regions (eight pixels in five LV slices; one slice per subject) were not significantly different when average λ values from each subject were used (p = 0.54) or when a single average λ value was used for all five subjects (p = 0.14).

Figure 4 and Figure 5 also reveal a trend that regions of tissue with more blood flow (during stress imaging, for example) are more likely to be underestimated than regions of low blood flow. This effect is partially caused by over-regularization of the higher flow data, which typically has a higher CNR. While perfusion estimates are relatively insensitive to small changes in CNR, in which an average λ value is suitable for model-independent analysis, large differences in CNR require different regularization weight parameters.

## Conclusion

This work demonstrates that a model-independent analysis method that uses iterative minimization and temporal regularization can be used to estimate myocardial perfusion in DCE-CMR studies. The method is flexible to accommodate different types of spatial or temporal regularization, which can reduce the effects of motion and noise in the blood and tissue enhancement data.

Estimates of myocardial perfusion using model-independent analysis are dependent on the choice of the regularization weight parameter, which increases nonlinearly to handle large decreases in the CNR of the measured tissue enhancement data. However, a fixed average λ value can give good results for fitting time curves with similar but not identical CNR values, such as found with pixelwise perfusion estimates.

Perfusion estimates with this method in five normal subjects imaged with 3 T CMR correlated with perfusion estimates from dynamic ^13^N-ammonia PET (y = 0.90x + 0.24, r = 0.85), a current standard for non-invasively quantifying myocardial perfusion. It must be noted however, that the results and conclusions drawn from this study are limited because of the small number of subjects imaged. Additional studies in normal and diseased patients are needed to further evaluate and validate the accuracy of this method.

## Competing interests

The authors declare that they have no competing interests.

## Authors' contributions

NP participated in the data acquisition, developed methods, performed data and statistical analysis and drafted the manuscript. ED conceived of the study and was involved in the data acquisition, method development, data analysis and editing. TR participated in part of the PET data acquisition and helped develop and perform the PET data analysis. DK developed the PET data analysis. CM participated in the design and coordination of the MRI and PET data acquisition. RB, PC, and JH assisted in the design and coordination of the PET imaging aspects. All authors read and approved the final manuscript.
